# A sensitive, stable, continuously rotating FFL MPI system for functional imaging of the rat brain

**DOI:** 10.18416/IJMPI.2022.2212001

**Published:** 2022-12-21

**Authors:** Eli Mattingly, Erica E. Mason, Konstantin Herb, Monika Śliwiak, John Drago, Matthias Graeser, Lawrence L. Wald

**Affiliations:** a Harvard-MIT Division of Health Sciences & Technology, Cambridge, MA, USA; b Martinos Center for Biomedical Imaging, Massachusetts General Hospital, Charlestown, MA, USA; c Harvard Medical School, Boston, MA, USA; d ETH Zurich, Department of Physics, Zurich, Switzerland; e Massachusetts Institute of Technology, Department of Electrical Engineering & Computer Science, Cambridge, MA, USA; f Fraunhofer Research Institution for Individualized and Cell-Based Medical Engineering (IMTE), Lübeck, Germany

## Abstract

Magnetic particle imaging noninvasively maps the distribution of superparamagnetic iron oxide nanoparticles with high sensitivity. Since the particles are confined to the blood pool within the brain, it may be well-suited for cerebral blood volume (CBV)-based functional neuroimaging with MPI (fMPI). Here, we present a magnetic particle imaging system designed to detect the CBV modulation at the hemodynamic timescale (~5 sec) in rodents. It has the capacity to record sufficiently fast image time-series for several hours continuously. The time-series imaging was achieved with an optimized drive coil that maintains ~0.01% per minute current magnitude stability. An electrical slip ring and rotary union for cooling water allows continuous mechanical rotation of the 2.83 T/m Field-Free Line (FFL) permanent magnets and shift coils. The system achieves a 6.7 ng Fe detection limit (SNR = 5) in a single 5 sec image in the time-series, a spatial resolution of 3.0 mm in a 3 cm diameter field of view. The designs have been made open-source to enable replication of this device.

## I. Introduction

MPI has excellent sensitivity, a quantifiable contrast source, and no biological background signal. These characteristics, together with the blood-pool origin of the cerebral signal of untargeted iron nanoparticle tracers, make it attractive for functional neuroimaging of cerebral blood volume (CBV) modulations. For example, cognitive tasks or pharmacological challenges are assessed by monitoring the signal intensity changes in an image time-series to infer functional activity. In proof-of-concept studies, we demonstrated high-contrast measurements of brain hemodynamic modulations in rats using non-localized MPI detection [1]. In order to provide full MPI time-series imaging of the rodent brain we have developed an FFL MPI system specifically designed toward fMPI applications which: i) images with a temporal resolution of under 5 seconds for at least 30 minutes, ii) undergoes minimal temporal drift during the 30-minute experiment, and iii) provides maximal sensitivity to SPION concentration changes without sacrificing the prior two goals.

### I.I. Design goals

#### I.I.1. Temporal resolution goal

Since hemodynamic changes associated with cognitive, sensory or pharmacological stimuli evolve on a timescale of ~5 sec, this represents the minimum temporal resolution needed for the application. This is based on data from functional magnetic resonance imaging (fMRI) studies showing the hemodynamic response function has a time constant of between 5 seconds [2] and ~20 seconds [3]. Given that a rotating FFL system can produce an image every half-rotation, this requires a gantry rotational velocity of 6 revolutions per minute, which is achievable with a stepper motor drive.

#### I.I.2. Minimum resolution and sensitivity goal

**Figure 1:**
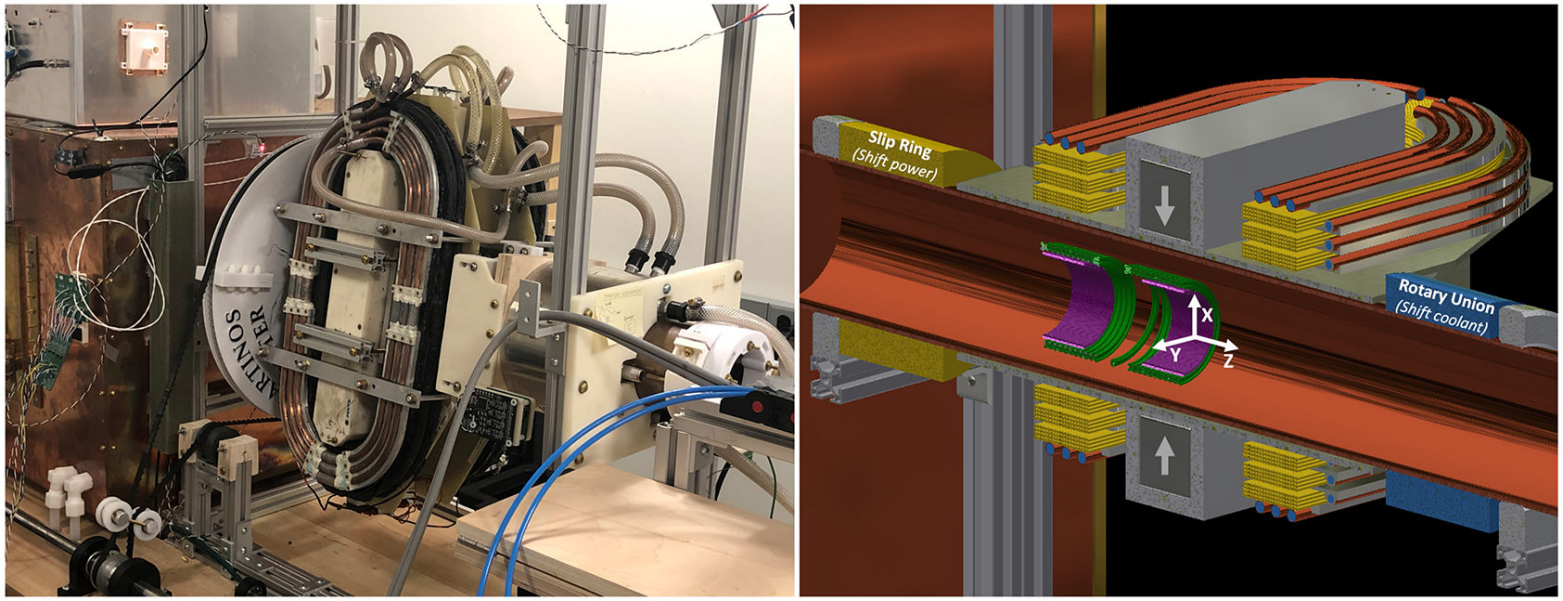
**Left**) A photograph of the MPI system. The amplifier racks containing the drive amplifier and shift amplifiers are out of view. **Right**) A cross-sectional rendering of the MPI system. In this rendering, the receive (“Rx”) coils are in purple, drive coils are in green, and shift coils and the slip ring are in yellow. The rotary union and water within the cooling tubes are in blue.

To achieve 3 mm intrinsic spatial resolution with practical SPION Langevin responses (full width half maximum (FWHM) of the susceptibility curve ≈ 4-5 mT), an FFL gradient of about 3 T/m is needed. For this we use the FWHM of the “normal” point spread function (PSF) [4] for the in-plane (X-Y in Figure 1) resolution, which is normal to the drive direction. This can be achieved with permanent rare-earth (NdFeB) magnet structures, which simplifies the power requirements for the continuously rotating gantry.

We define the minimum sensitivity goal for the imager as the ability to detect a 5% change in the rat’s cerebral blood volume within a single voxel (3 mm x 3 mm x 3 mm) in a single 5 sec image with a signal-to-noise ratio (SNR) of 1. Functional CBV changes are expected to be about 4x this [3]. The resting Fe concentration in the rat’s cerebral blood supply has been estimated to be 200 ng in a (3 mm x 3 mm x 3 mm) voxel after a 10 mg/kg dose [1]. A 5% Fe change from activation-induced CBV change corresponds to delta of about 10 ng Fe in the voxel. If, for instance, this change in voxel Fe is seen with SNR = 1 in a single image in the time-series, then over the course of a 360 image time-series (30 min) with multiple activation blocks, the statistics of the detection will improve with the square root of the acquisition time for additional factor of ~20x (assuming uncorrelated noise).

#### I.I.3. Stability goal

Temporal intensity drift becomes especially important for time-series imaging since the interpretation of the data relies on temporal stability, and the sample cannot be frequently removed to acquire a new baseline. While some background drift in image intensity over time is inevitable, full mitigation of this drift is challenging and still an active area of research [5]. Our stability goal for a 30-minute experiment is to reduce temporal signal drift of the resting blood volume signal to below that of the thermal noise level of the time-series images. If the system exactly meets this sensitivity goal, the signal can drift no more than 5% (our sensitivity goal) in that time. In MPI systems, signal is roughly proportional to drive current [6]; this dictates a drive current stability goal of about 5% peak-to-peak over 30 minutes.

For phase drift, a small-angle approximation (sin*θ* ≈ *θ*) dictates a maximum peak-to-peak phase drift of 0.05 radians.

#### I.I.4. Field of view goal

To image a rat brain *in vivo,* the imaging volume should be about 30 mm diameter and about 40 mm in length. For time-series imaging, transverse 2D images of the brain are sufficient, and the 3D images are only necessary for anatomy and localization. 3D images are accomplished via mechanical movement of the bed. The free bore must be substantially larger, at least 50 mm diameter to accommodate the bed, breathing tubes, ears, etc.

## II. Methods and design

### II.I. Imager configuration

The system is photographed and depicted in cross-section in Figure 1. The imager is based on a mechanically rotated permanent-magnet generated FFL which is shifted by water-cooled electromagnet shift coils. The drive solenoid and receive coil solenoidal gradiometer pair are stationary inside a cylindrical copper bore tube (shield) which is also stationary. This configuration was chosen to take advantage of manufacturing simplicity, the sensitivity benefits of FFL-based imaging [7], and the efficiency of the solenoidal drive coil. The hardware configuration is similar to a previously described imager [8].

To achieve the 5 second temporal resolution goal, a slip ring (SR007, Rotary Systems, MN, USA) and rotary union (MEPH400-04ID113, MOFLON, Shenzhen, CN) were necessary for continuous rotation. Otherwise the gantry would need to reverse directions periodically to prevent the shift coil current supply wires and cooling tubes from wrapping around the frame. Similarly, rapidly stopping and reversing the gantry’s direction would be impractical. Alternatively, an electrically rotating FFL system architecture [7, 9-11] or a multi-mode rotating Halbach configuration [12] could be feasible. Other approaches have included rotating the sample [8, 13] instead of the FFL magnets and shift coils, but this is impractical with *in vivo* neuroimaging experiments.

### II.II. Gantry rotation

The addition of the rotary union dramatically increases rotational friction (to ~45 Nm) which must be overcome by the motor and mechanical gearing configuration. We utilized a powerful stepper motor (42Y312D-LW8, Anaheim Automation, CA, USA), operated at 112.5 RPM with a belt-drive system providing an 18.75-fold total mechanical reduction to reduce the rotational speed to 6.0 RPM (10 sec per rotation, yielding an image every 5 sec). To achieve the mechanical reduction we use timing belts with an 18 teeth to 40 teeth pulley followed by an 18 tooth pulley driving a 152 tooth pulley fabricated by a 3D printer (the 470 mm dia. white disc in Figure 1).

To keep track of the gantry position, an 8-bit absolute encoder (EAW0J-B24-BE0128, Bourns Inc., CA, USA) is coupled to the small shaft prior to the final mechanical reduction. Thus the effective resolution is increased 8.44x yielding ~0.2 deg of gantry rotation per bit. This is supplemented by recordings from two homing switches that are ~180 degrees apart on the gantry. Thus there is a "home" switch event marking the absolute gantry position during each image. We utilize integral control feedback to the motor speed controller to limit error propagation in gantry rotation over long experiments. Further, the imaging sequence can be triggered on one of the home switches for a consistent start location.

### II.III. Field-free line magnets and shift coils

#### FFL magnets:

The FFL is generated by two 50.8 mm x 50.8 mm x 406.4 mm N48 NdFeB blocks magnetized in the 50.8 mm direction (see Figure 1). They are mounted 12.7 cm apart with their magnetization anti-parallel. We measured the field gradient using two Hall-effect sensors (SS39ET, Honeywell, Charlotte, NC) spaced by 1.5 cm.

#### Shift coils:

**Figure 2:**
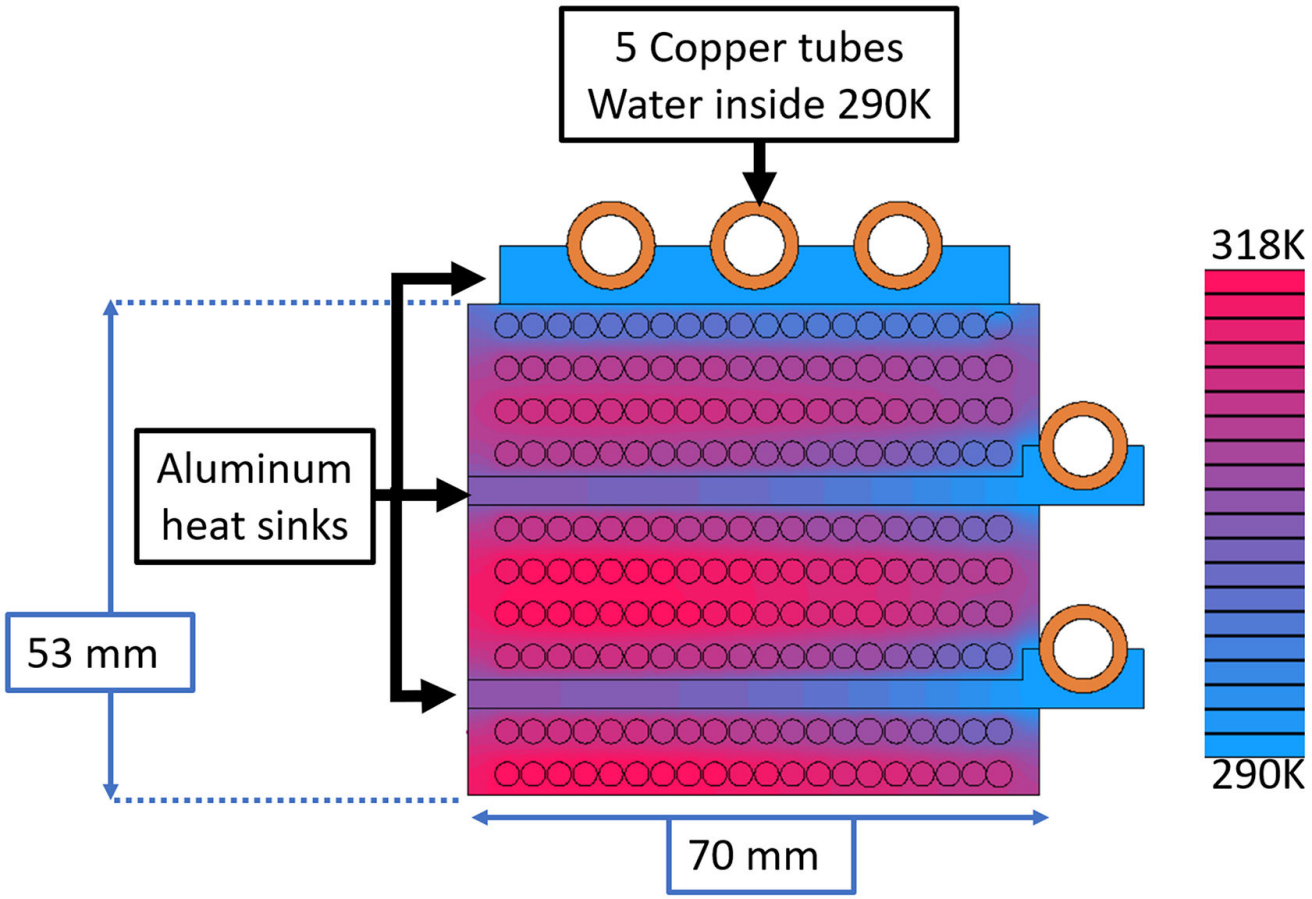
Configuration and thermal analysis of the shift coil in cross-section. Thermal modeling assumed 42.5 Amps peak, which corresponds to a FOV of 30 mm. The thermal conductivity of the epoxy matrix around the wires is modeled with a thermal conductivity of 0.3 W(mK)^−1^. The system boundary conditions were modeled as fully insulating besides the inside of the copper tubes, which were all assumed to be a fixed temperature of 290 K.

The shift coils modulate the position of the FFL at 2.7 Hz to form 27 projections per 5 sec image (half rotation of gantry) providing the 5 sec image temporal resolution. An 84 mT peak-to-peak shift field amplitude is required to move the FFL 30 mm (the image FOV). This is achieved with an approximate Helmholtz configuration of "racetrack" coils as seen in Figure 1. Each of the two racetrack shift coils contains 200 turns of 10 AWG magnet wire with three cooling heat sinks. These cooling plates were broken up in quarters to mitigate eddy currents. Figure 2 shows the winding and heat-sink configuration as well as the simulated thermal steady-state at 42.5 A peak which corresponds to a heat load of 1.6 kW. They operate in current control mode.

Table 1 shows parameters (resistance, inductance, mutual inductance and efficiency) of each of the shift coils after assembly on the gantry as measured with an in-house auto-balancing bridge impedance analyzer near the operating frequency (3 Hz) as well as the simulated parameters (FEMM 4.2 [14]).

### II.IV. Drive field chain

#### Power amplifier and transformer:

**Figure 3:**
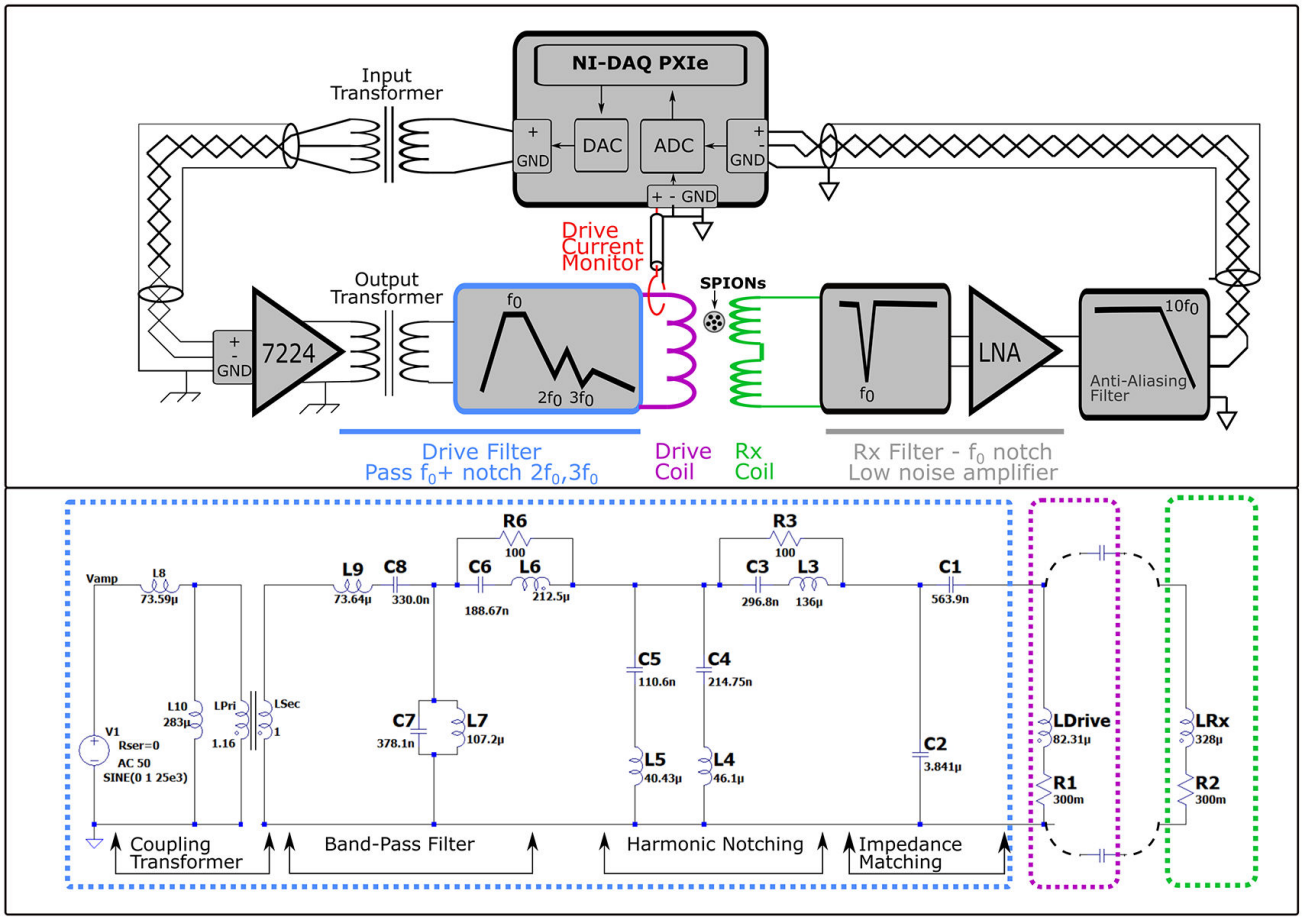
**Top**: Simplified schematic of the drive/receive system depicting the arrangement of amplifiers, filters, and wiring. **Bottom**: The LTspice (Analog Devices) model for the drive filter, coil, and coupling to the Rx coil.

The 25 kHz drive field current was generated by a power amplifier (AE Techron 7224, Elkhart, IN) which was found to have lower harmonic distortions and noise than the AE Techron 7548. The amplifier is operated with a transformer-coupled input and output to prevent ground-loops in the system as seen in Figure 3.

The input transformer is a toroid (B64290L0618X087, TDK Lambda, Tokyo, JP, N87 material, ID = 14.8 mm, OD = 25.3 mm, B_sat_ ≈ 500 mT), acting as a balun to convert the unbalanced output of the DAQ to a balanced input to the drive amplifier. This transformer was mounted in a two-layer shielded enclosure (inner material = aluminum, outer = steel) and ~1 m away from the rotating gantry to prevent the shift coils and permanent magnet rotation from modulating the core magnetization and inducing harmonic distortion in the drive waveform. The core flux density as a function of the transformer’s primary voltage is:

(1) $$B_{\text{pk}}=\frac{V_{\text{pk}}}{2\pi fNA_{e}}$$

where N is the turn count, and $A_{e}$ is the effective area of the core. We sought to keep the core flux density below 1% of saturation. This was achieved with N = 44 turns.

The output transformer consists of a pair of planar "E" shaped cores (B66297G0000X187, TDK Lambda, Tokyo, JP, N87 material) with similar design criteria. The two core halves are separated by a plastic spacer. This lowers the effective permeability of the transformer core, rendering it less sensitive to external effects (e.g. thermal or external DC magnetic fields that may partially saturate the core) to improve the drive chain stability and linearity. The gap also provides a degree of freedom that can be used to tune the leakage inductance and therefore fine-tune the drive filter. Following tuning, the transformer was potted in epoxy to prevent the gap size from changing. The core losses are negligible at the low operating frequency and flux level.

#### Drive filter:

Figure 3 shows the drive filter circuit details. The filter is a transformer-coupled, band-pass filter with harmonic notching. It differs from the balanced topology previously presented [15] in that it is unbalanced to reduce space and complexity.

The filter is designed around the impedance of the drive coil. In the filter, C1 and C2 determine the impedance transformation ratio (similar to ref. [16]). This C1, C2 impedance transformation, together with the 1:1 transformation of the amplifier output transformer, ensures the amplifier sees a load impedance that minimizes distortions (2-8 Ohm and near zero reactive impedance). Second, by using C1 and C2 to step up the impedance by a factor of m the current being carried by the series sections in the filter is reduced by roughly $\sqrt{m}$, thereby reducing the power dissipated by approximately m. This reduces heating and thermal drift, making passive cooling sufficient.

To further mitigate the effect of thermal drift (dominated by the capacitors’ temperature coefficient), the filter was designed with its transconductance (output current per input voltage) operating point at a local minimum for the magnitude, and in a relatively flat response region for phase (see Supplemental Data).

The filter’s transconductance was measured by using a Rogowski coil current monitor [17] and variable-gain pre-amplifier (Model 5113, Ametek, PA, USA). The transfer function was measured in multiple overlapping sections and stitched together accounting for the different gains. The inherent differentiation that the Rogowski coil adds to the measurement was accounted for in the data post-processing. The Rogowski coil itself was calibrated against a Hall effect current monitor (ACS-770-050B, Allegro MicroSystems, New Hampshire, USA).

Each individual filter component was measured in place (both leads disconnected from the circuit) with the auto-balancing bridge impedance analyzer at the drive frequency. Capacitors were characterized by a parallel capacitance and parallel resistance; inductors were characterized by a series inductor and resistor. Besides the transformer, all the inductors are air-core, and the primary capacitors are Celem CSM Nano (Celem, Jerusalem, Israel) with welded copper tabs to ensure the connections are linear, low impedance, and reliable. We observed increased noise and distortion with screw-terminal connections. The resonant pair was tuned to the drive frequency with high voltage, parallel small (<1 nF) polypropylene film capacitors (e.g. KEMET R75H series, 2 kV DC rated).

#### Drive coil:

**Figure 4:**
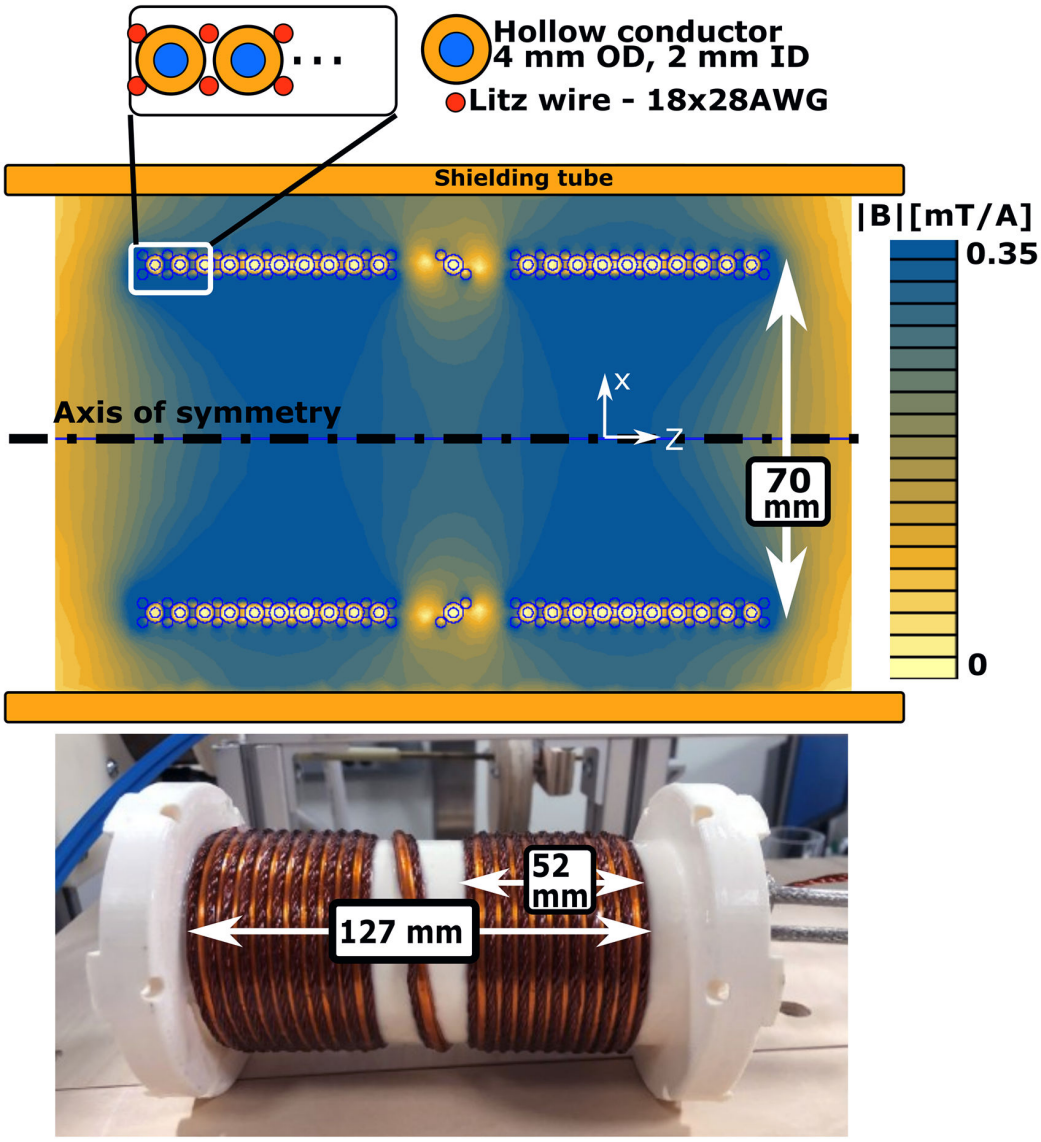
**Top**: 2D axisymmetric simulation of magnetic field and detail of winding pattern. The simulation was done in FEMM 4.2, and the simulation frequency was 25 kHz. The hollow conductor was modeled explicitly, and Litz wire was 18 strands of 28 AWG wire in a 2.1 mm diameter circle. **Bottom**: Photograph of the coil outside of the bore. Note the entire coil, including inside layers, is saturated with epoxy (Loctite 608, Loctite, CT, USA).

Figure 4 shows the drive coil winding pattern, field simulation, and photograph. The coil is designed to produce a minimum of 8 mT pk in the center of the FFL at 100% duty cycle. This amplitude was selected as a balance between signal level and thermal limits.

The winding pattern for the drive coil (Figure 4, top) is a three-layer design with the inner and outer layers being ~2 mm diameter in-house made Litz wire (4 twisted bundles consisting of 2 bundles of 4 strands and 2 bundles of 5 strands for an effective 18/28AWG). This wire was chosen over available commercial alternatives, which were covered with a PFA shell, to ensure epoxy fully penetrated the strands. The epoxy impregnation stabilizes the strands and improves thermal contact (thermal conductivity of epoxy ≈ 10x thermal conductivity of air). The middle layer of turns is composed of 4 mm outer diameter (OD), 2 mm inner diameter (ID) copper tubing (polyimide tape insulated). The three layers (Litz, tubing, and then Litz) are wired in series, so the copper tubing also carries current and thus contributes to the field, in addition to water flow through the inner channel. This design allows for excellent thermal contact between the Litz wire and the cooled tubing. The closeness of these wires results in eddy current losses in the solid wire (proximity effect), and at 25 Amps peak (8 mT peak at FFL) this proximity loss (eddy current loss in tubing from Litz wires) is 25 Watts. These additional losses slightly increase the cooling water and amplifier power requirements.

The shielding tube between the drive coils and the rotating gantry/shift coils is 3.1 mm-thick copper, and extends over 100 mm past the end of each coil. The length and thickness allow for excellent shielding (skin depth at 25 kHz ≈ 0.4 mm). The tube also reduces the efficiency of the coil due to eddy currents generated within the copper. This motivates keeping as much space between the transmit coil and the shielding tube as possible.

The drive coil field was verified by using a SS39ET hall effect sensor (bandwidth = 50 kHz) located in the center of the bore at the FFL location.

#### Wire selection and water coolant:

Chilled water is circulated to keep the drive coil and rodent bore temperatures low. The 1 mm wall-thickness hollow conductor of the drive coil provides about 2 skin-depths for current flow (skin depth ~0.4 mm at 25 kHz) and was readily commercially available. Proximity simulations were run using FEMM 4.2 [14]. The expected water flow rate was 5 mL/s as determined from the pump output pressure (approx. 20 PSI (138 kPa)), the tube length (4.6 m), and inner diameter (2 mm) by using Eqn. 13 in Ref. [18].

With this flow rate, the thermal calculations predict that the 100 Watts generated by the drive current will cause the water to reach a steady-state temperature of ~5 °C above the input water temperature. This leaves significant flexibility in case the pump underperforms, junctions cause viscous losses, etc.

#### Thermal simulations:

A steady-state thermal analysis was performed in FEMM 4.2 to determine the temperature distribution within the drive coil compartments (Litz wire, epoxy and water coolant tubes). The analysis was a 2D axisymmetric simulation, and the power generation was defined from the electromagnetic simulations. The hollow conductor’s volumetric heat generation was the mean loss density within the copper: 0.60 MWm^−3^. Similarly, the inner and outer Litz wire layers were given power densities from the electromagnetic simulation of 1.16 MWm^−3^ and 1.13 MWm^−3^, respectively. For simplicity, the water temperature was fixed at 300 K across the full coil (rather than varying by 5 °C from input to output).

#### Electromagnetic simulations:

The electromagnetic simulations were done in FEMM 4.2 using a 2D axisymmetric model at 25 kHz. The hollow conductor wire was modeled as the default copper, and the Litz was modeled using the Litz wire option with 18 strands of 28 AWG wire. The simulated and measured inductance, resistance and coil sensitivity is found in Table 2.

### II.V. Received signal chain

#### II.V.1. Receive coil

The receive coil is a two-part gradiometer design coaxial with the drive coil. One half of the Rx coil is wound parallel to the drive coil solenoid and the other is wound anti-parallel, thus approximately cancelling mutual inductance with the drive coil. The receive coil is two layers (33 turns inner, 32 turns outer) and each of the two sections is 4 cm long. The inner clear-diameter (54 mm) was selected to be as close as possible to the rat’s head to maximize sensitivity.

To adjust the gradiometer to minimize the induced voltage from the drive field (“feedthrough”) we temporarily glue a ~50 cm fiberglass rod threaded through a fixed captive nut and mechanically adjust the relative positions of the drive and Rx coil while monitoring the feedthrough. Once a minimum voltage is found, the Rx coil is removed, coated in epoxy (Loctite 608, Loctite, CT, USA), and the procedure is repeated as the epoxy sets. The epoxy ensures the coil pair maintains their relative position and is not sensitive to the mechanical vibration generated by the mechanically rotating system.

**Figure 5:**
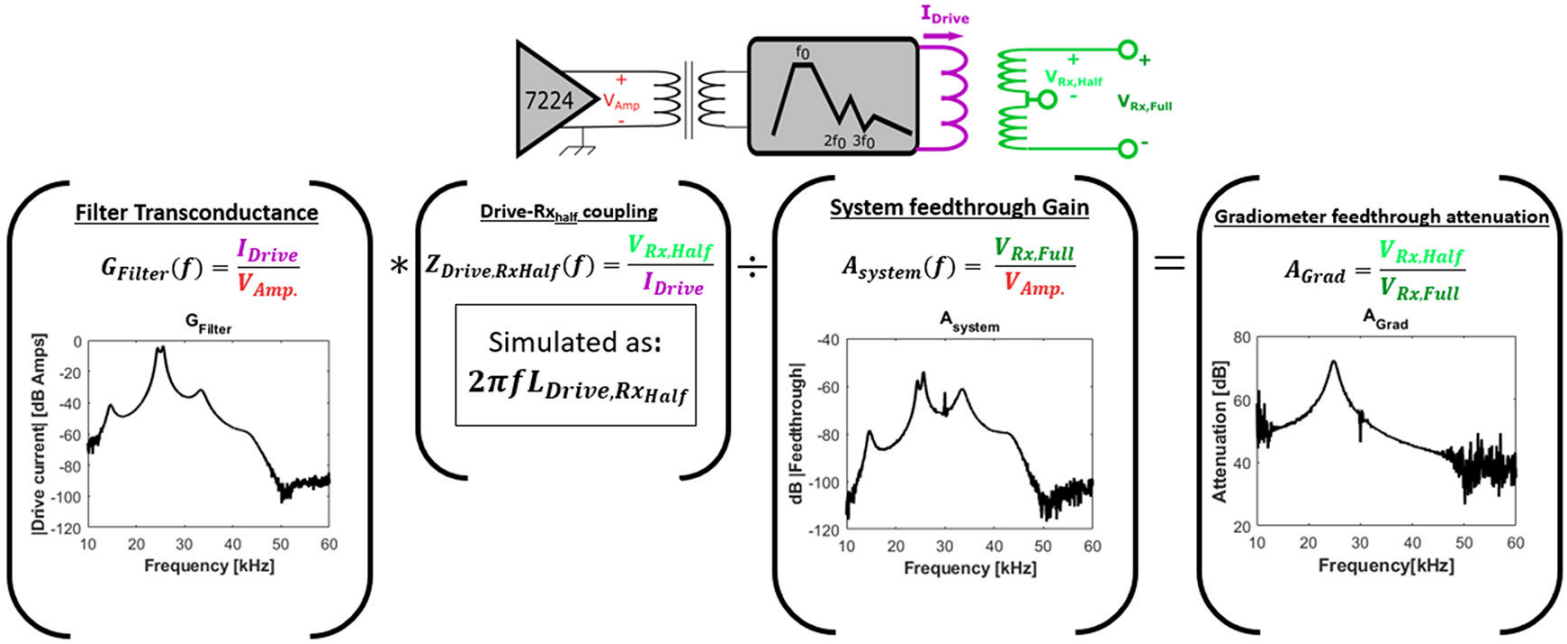
The gradiometer feedthrough attenuation and intermediate measurements. The transconductance of the filter (G_Filter_) is the drive coil Amp per Volt from the amplifier. The Drive — Rx_Half_ coupling (Z_Drive,Rx_Half__) is the voltage across half of the Rx coil gradiometer per Amp in the drive coil, and is simulated with FEMM 4.2 at 25 kHz and assumed to be linear with frequency. The total system feedthrough gain (A_System_) is the voltage across the full Rx coil per Volt to the drive filter from the amplifier. The gradiometer feedthrough attenuation is G_Filter_ * Z_Drive,Rx_Half__/A_System_. This represents the effectiveness of the gradiometer configuration at suppressing pickup of the drive coil signal.

The “Gradiometer feedthrough attenuation” ($A_{\text{Grad}}$) in Volts per Volt, is defined as:

(2) $$A_{\text{Grad}}(f)=\frac{V_{\text{Rx},\text{Half}}(f)}{V_{\text{Rx},\text{Full}}(f)}$$

where $V_{\text{Rx},\text{Half}}$ is the voltage per Amp which the drive coil induces in either of the two halves of the gradiometer, and $V_{\text{Rx},\text{Full}}$ is the total voltage across the gradiometer per Amp in the drive coil. $V_{\text{Rx},\text{Full}}$ is straightforward to measure by measuring both the drive coil current per input Volt to the filter (Filter transconductance, $G_{\text{Filter}}$) and the full gradiometer voltage per input Volt to the drive filter (the total system feedthrough gain, $A_{\text{System}}$), and taking their ratio. $V_{\text{Rx},\text{Half}}$ is not easily acquired because the gradiometer is wound with a single continuous Litz wire. $V_{\text{Rx},\text{Half}}$ is taken from simulations done in FEMM 4.2. This is simulated only at the drive frequency, and a linear frequency dependence is assumed. This is illustrated in Figure 5.

#### II.V.2. Rx filter and preamp

**Figure 6:**
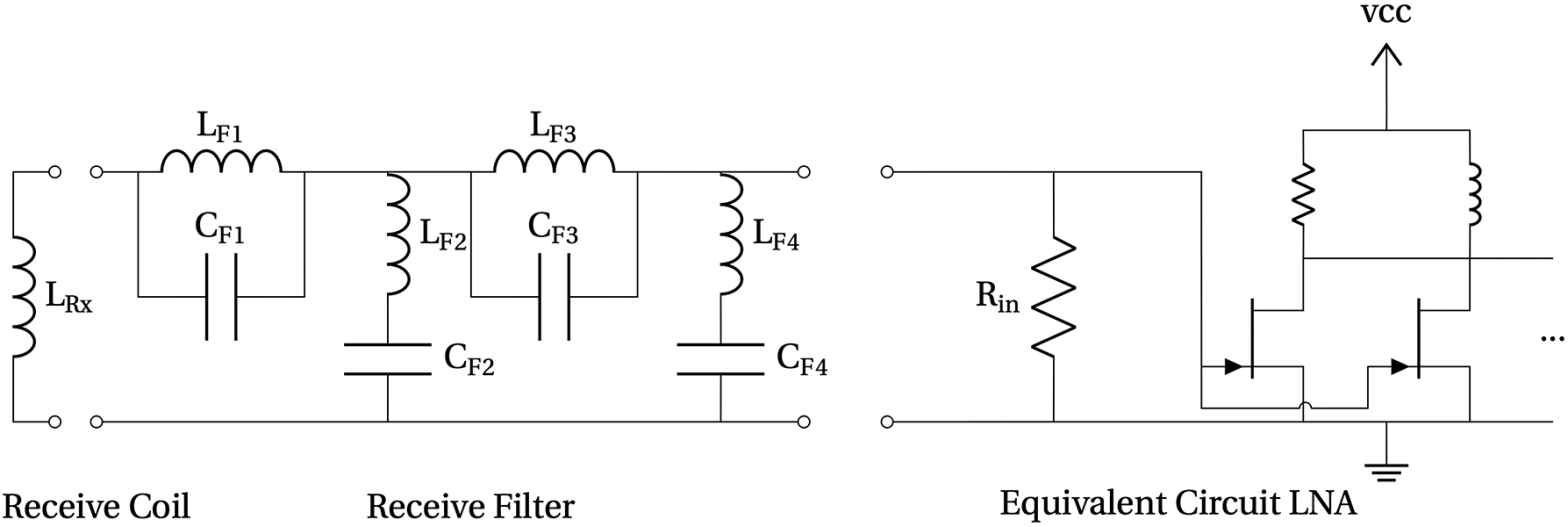
Circuit model of the receive filter and the equivalent input circuit of the LNA.

In order to suppress remaining feedthrough, the receive signal is filtered by a 4^th^ order resonant notch filter, as seen in Figure 6. Resonant filters have the advantage of a very high suppression at the resonant frequency and very low noise contribution at the desired higher harmonics. The receive filter consists of series and parallel LCs, for which the parallel sections (e.g. L_F1_, C_F1_) maximize impedance at resonance and the series sections (e.g. L_F2_, C_Fc_) minimize impedance at resonance, forming voltage dividers. The filter performance, therefore, depends on the effective impedance of the parallel sections at resonance, $R_{p}=\frac{L}{C\cdot R_{s}}$, with $R_{s}$ being the overall resistance within the resonant circuit, C being the capacitance and L being the inductance. From this equation, one can see that this effective resistance at resonance can be maximized by choosing the inductance L as high as possible, keeping the power consumption in mind. Another possibility is to choose an inductor design that maximizes the quality factor of the coil and keeps $R_{s}$ small for a desired L.

For the serial circuit (e.g. L_F2_, C_F2_) the impedance at resonance is only dominated by the resistance of the circuit. Therefore, this resistance should be minimized under the condition that the series section inductors (L_F2_∥L_F4_) are significantly larger than the receive coil. This is because at frequencies above the resonance there would be a voltage division between L_Rx_ and L_F2_∥L_F4_.

In the used filter, the first two stages are air core coils. This is because the first stage (L_F1_, C_F1_) has to handle nearly the full voltage of the receive coil at the drive field frequency, and it may carry large currents depending on the cancellation and stability of the gradiometer. A ferrite core coil may be driven in a nonlinear region and generate unwanted harmonics. The second stage (L_F2_, C_F2_) is also designed using air core coils. Both inductor quality factors are maximized using a toroid cross-section matching optimal inductor shapes [19]. As the voltage after the second stage is already in the region of a few mV, the inductors in the following stages are designed using ferrite cores (B64290, TDK) in order to reduce size and boost the quality factor.

After filtering, the signal is connected to a JFET input (2SK2394-7, Onsemi, Pheonix, AZ) low noise amplifier. The amplifier has three amplification stages. The first consists of 20 2SK2394-7 JFETs in a parallelized common source configuration (forward transconductance = 38 mS each, 760 mS total. Gain ≈ 25). As the input signals are only in the range of a few mV the input bias circuitry and working point resistors are neglected in order to improve the noise output [20, 21]. In order to limit power dissipation of the drain resistors, the drain resistor (33 Ω) is connected in parallel to an inductor (2 mH, self-wound on ferrite core B64290, TDK). This effectively bypasses the DC current from the resistors but preserves the impedance in the operational frequencies, and allows the JFETs to be in saturation. The second and third stages are designed using a non-inverting operation amplifier circuit with a dual low noise, low distortion, high speed ICs (ADA4898, Analog Devices). The output of the LNA is made differential by a fully differential operational amplifier (AD8138, Analog Devices) to reduce interference picked up between the LNA and data acquisition, and then further put through a fully differential anti-aliasing filter (cutoff = 250 kHz).

In total the filter provides a very high suppression at the fundamental frequency and a low noise signal processing and amplification for the desired receive band, which in total lead to the high sensitivity of the overall system.

As the resonant receive chain and the LNA result in a frequency dependent amplification and phase, the recorded signal in time domain does not simply reflect an amplified version of the voltage induced in the receive coil. To correct this alteration of the signal, the complex transfer function is recorded and applied to the digitized signals. To measure the transfer function of the receive chain, a test coil on a printed circuit board is swept in frequency and a 50 Ω series resistor is used to measure the current in the coil. The receive chain is then defined as:

(3) $$T_{\text{RX}}(f)=\frac{V_{\text{Out}}(f)}{N_{c}\cdot I_{c}(f)\cdot A_{c}},$$

where $T_{\text{RX}}$ is the receive chain transfer function with dimensions of $\frac{V}{Am^{2}}$, $V_{\text{Out}}$ is the receive chain output voltage in Volts, $N_{c}$ is the turns on the test coil and is dimensionless, $I_{c}$ is the current in the test coil in Amperes, and $A_{c}$ is the cross-sectional area of the test coil in m^2^.

##### Data acquisition, transmission, and reconstruction:

We utilize NI-DAQ-PXIe 6363 (National Instruments, Austin, TX) inputs exclusively for the differential measurement of the Rx coil signal sampled at 1 MHz. We simultaneously receive the drive and shift current measurements using a secondary independent DAQ, the NI-DAQ-PXIe 6361. By this, the SPION detection signal (Rx) and the image encoding signals (shift/drive) are completely separate. This eliminates any residual channel-channel crosstalk.

The whole imaging system is controlled by a home-built custom acquisition software implemented in LabVIEW (National Instruments, Austin, TX). Synchronization of acquisition tasks is performed by the DAQ system with negligible jitter and delay times. Our fully parallelized console software subdivides the sampled wave-forms on-the-fly into waveform segments of 2.8 ms. On each segment, we perform a Fast Fourier Transform (FFT) and extract amplitude and phase values of the harmonics of the fundamental drive frequency.

By binning these data points into projection-space coordinates (i.e. build sinogram where each projection is a column), we can achieve a real-time reconstruction of the image by using a simple inverse Radon (“IRadon”) reconstruction. This simple IRadon reconstruction uses only the 3f_0_ signal component and performs a complex affine baseline subtraction using the first and last N points from each projection. This assumes there is no sample outside of the FOV. All images presented here utilize this reconstruction technique.

The parallelized system design ensures that actual data acquisition is not slowed down by data processing of the host computer. In principle, our software architecture is also capable of running more advanced reconstruction algorithms in real time. Imaging parameters as well as reconstruction parameters can be adjusted during runtime.

#### II.VI. System performance measures

##### II.VI.1. Drift and stability

To assess the temporal stability we imaged a 4 *μ*L phantom with 250 ng Fe sample in the center of the field of view continuously for 30 minutes. Each 5.0 sec image utilized an 8 mT peak drive field at 25 kHz. The drive and shift coils were cooled as described in their respective sections, and no other components were actively cooled or actively temperature compensated. During this image sequence, no feedback control on the current magnitude or phase was used. With these data, two measures of stability were assessed: 1) The drift in the magnitude and phase of the drive coil current over this time, and 2) the image signal amplitude as a function of time. For typical imaging experiments, 5 images are taken continuously per set with a half rotation (5 sec) gap such that the next set can re-trigger on the gantry home switch yielding 83% duty cycle.

##### II.VI.2. Sensitivity

To determine the imager’s sensitivity (minimum detectable mass given a signal-to-noise ratio of 5), we acquired 60 5-second images and computed the peak SNR (mean and standard deviation) of the images for each concentration in a dilution series of Synomag-D (Lot:16321104-02, Micromod Partikeltechnologie GmbH, Germany). Concentrations used were: 6 mg Fe/mL (undiluted), 0.5, 0.25, 0.125, 0.0625, 0.0313, 0.0156, 0.00781, 0.00391, 0.00195, 0.000977 mg Fe/mL). Each sample was a 4 *μ*L volume at the apex of a 0.25 mL microcentrifuge tube, thus the iron masses of the samples were: 24 *μ*g, 2 *μ*g, 1 *μ*g, 500 ng, 250 ng, 125 ng, 62.5 ng, 31.3 ng, 15.6 ng, 7.8 ng, 3.9 ng. Following image reconstruction onto a finer spatial grid than the instruments resolution, the images were smoothed to approximately that of the native instrumental spatial resolution by applying a 3x3 pixel (1.3 mm x 1.3 mm) flat-top smoothing kernel. In each of the images, the signal-to-noise ratio (SNR) was defined as the signal in the center of sample divided by the standard deviation of the noise within an empty-bore image. We plotted the mean image SNR versus mass, and fitted a line constrained to cross at the origin to determine the detection limit (the concentration expected to yield SNR=5) for these SPIONs.

To determine the imager’s noise floor in terms of detectable magnetic moment, we acquired 100 24-ms empty bore signals. The RMS power spectral density of each acquisition ($\sqrt{{\text{PSD}}_{V}}$) in units of $\frac{V}{\sqrt{Hz}}$ is computed and normalized by $T_{\text{RX}}(f)$ for these measurements. The system noise floor ($S_{\text{Noise}}$) in $\frac{Am^{2}}{\sqrt{Hz}}$ is the standard deviation of this quantity:

(4) $$S_{\text{Noise}}=\text{std}\left(\frac{\sqrt{{\text{PSD}}_{V}}}{T_{\text{Rx}}}\right)$$

##### II.VI.3. Spatial resolution

The in-plane (X-Y) spatial resolution is defined as the full width at half maximum (FWHM) of the system’s point spread function. We measure this by imaging a known SPION distribution (1.75 mm circle) and minimizing the error between the ground truth object convolved with a Gaussian kernel and the reconstructed image. The best fitting Gaussian’s FWHM is considered to be the spatial resolution.

## III. Results

### III.I. Drive field chain

The measured transconductance of the filter (drive coil Amp per Volt from the amplifier) is seen in Figure 5(left) and in Supplemental Data. At the drive frequency, the transconductance is 0.4 Amps in the drive coil per Volt into the filter, and the second and third harmonic are attenuated by 94 dB and 127 dB respectively (using simulated values because the measured values have substantial noise due to dynamic range limitations). The system feedthrough, which is the voltage across the Rx coil per Volt input to the drive filter, is shown in Figure 5 (center). The 2nd harmonic feedthrough value ($A_{System}$) is about −108 dB, though quite noisy due to dynamic range limitations.

The measured field per Amp at the center of the bore is 0.34 mT per Amp. The temperature of the output water at steady state is ~ 9 °C above the input, and the interior surface of the Rx coils (i.e. where the sample resides) reaches a steady-state temperature of ~ 5 – 10 °C above the input water temperature at steady-state operation (depending on airflow, location, etc.).

### III.II. Receive signal chain

**Figure 7:**
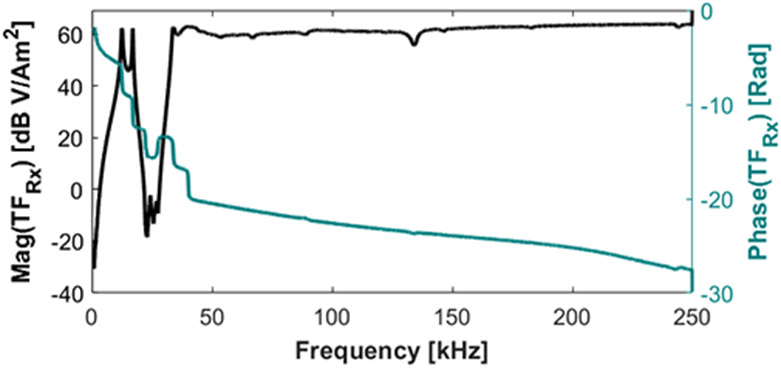
Transfer function between a test coil in the center of the receive coil (at the location of the FFL) and the output voltage.

**Figure 8:**
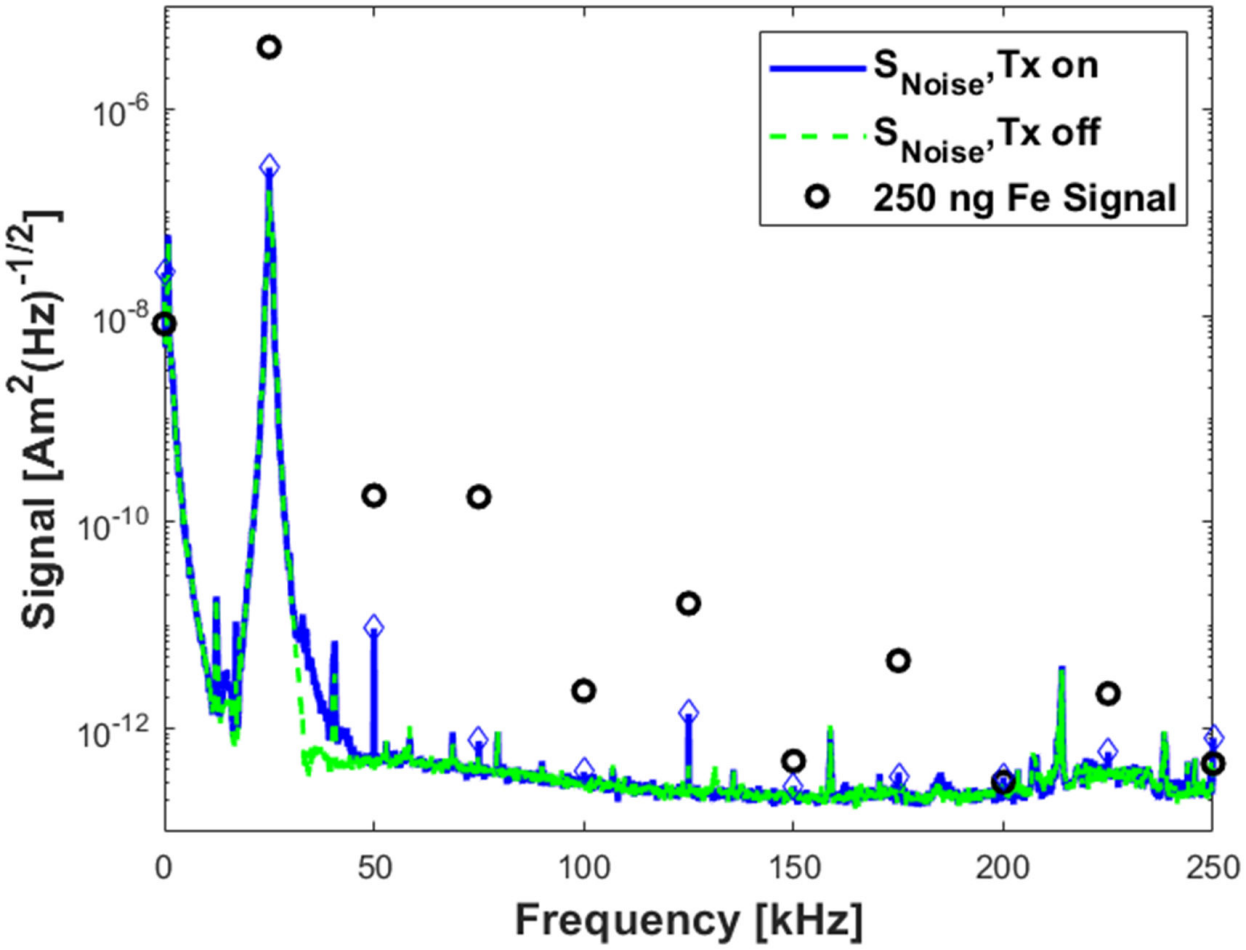
Spectral density of imager noise ($S_{\text{Noise}}$), with the drive amplifier on (blue line, blue diamonds at harmonics) and off (green dashed line), and signal from 250 ng Synomag-D (black open circles), all referred back to magnetic moment at the coil. $S_{\text{Noise}}$ is the standard deviation of each frequency component in the power spectral density (in Am^2^ * Hz^−1/2^) from 100 different 24 ms acquisitions. This shows the baseline noise of the system.

At the drive frequency the gradiometer feedthrough attenuation is 71.5 dB, but decreases to about 40 dB by the second harmonic, and decreases thereafter. The receive chain transfer function from magnetic moment at the FFL to output voltage is shown in Figure 7. Figure 8 shows the baseline imager noise, $S_{\text{Noise}}$, with the drive coils powered, referred to magnetic moment. Here, the blue diamonds indicate the relevant harmonics. The open circles show the signal strength from a 250 ng Fe sample. The noise level of the 2nd and 3rd harmonics with the drive on (equal to 9.4 and 0.7 ${\text{pAm}}^{2}∕\sqrt{\text{Hz}}$) are above the baseline noise level. This represents noise dominance from fluctuations in the unfiltered drive amplifier harmonics, or harmonics generated within the filter (e.g. capacitor distortion [22]).

### III.III. Field-free line and shift coil

The measured shift coil field per Amp is 1.0 mT, and using a 30 mm diameter field of view (42 Amps peak triangle waveform), the shift coils can operate for at least 2.5 hours at 100% duty cycle. The measured FFL gradient was 2.6 T/m in Z and 2.8 T/m in X. For Synomag-D 70nm particles (Lot:16321104-02, measured 4.2 mT FWHM response curve) this yields a nominal in-plane spatial resolution of 3.45 mm (accounting for the 2.3x factor from imaging in the ‘normal direction’).

### III.IV. System performance and imaging

**Figure 9:**
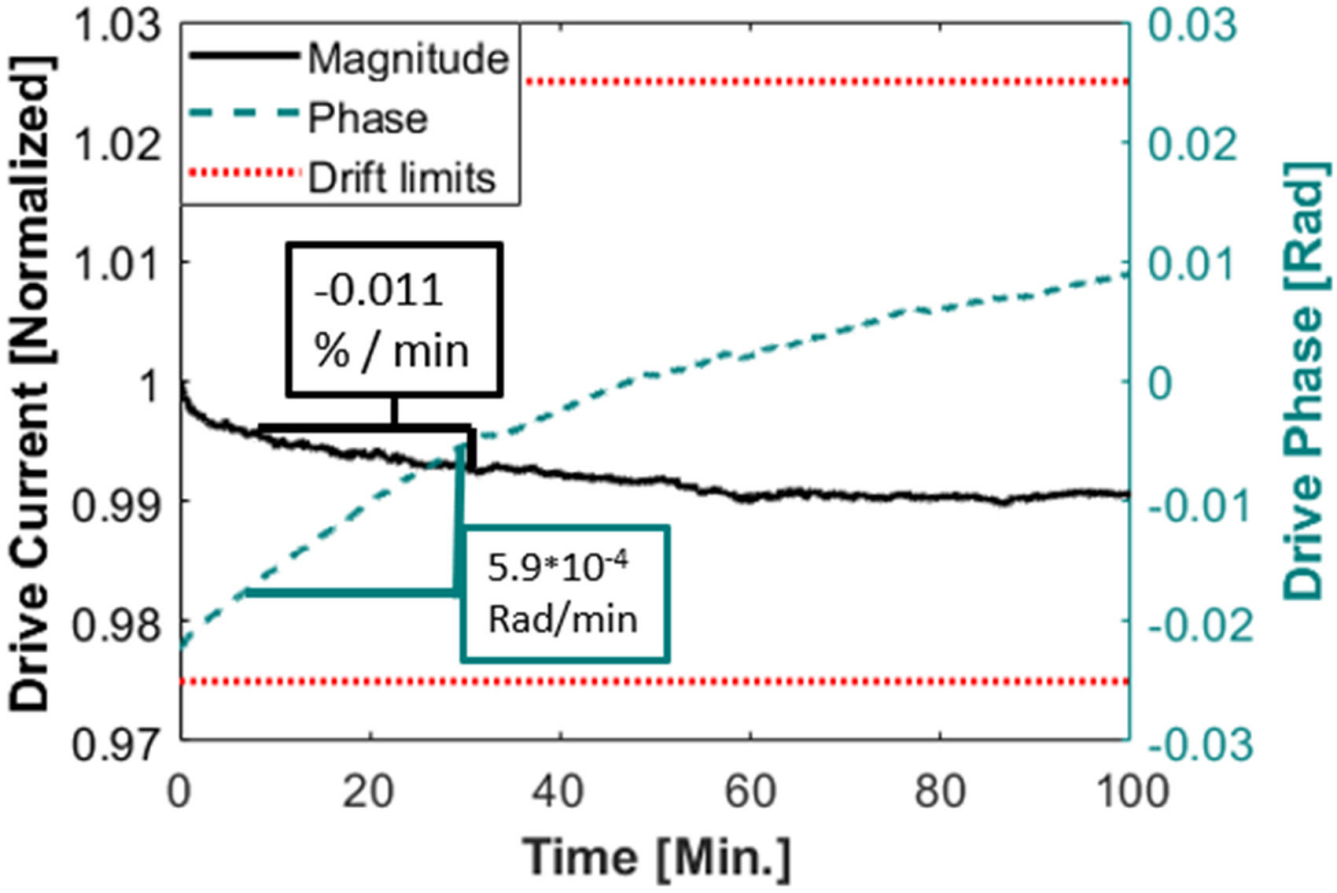
Drive current and phase drift over 30 minutes starting from cold as measured with an inductive (Rogowski coil) current monitor. The imager is operating at 100% duty cycle. There is no feedback control on the drive coil current or phase.

**Figure 10:**
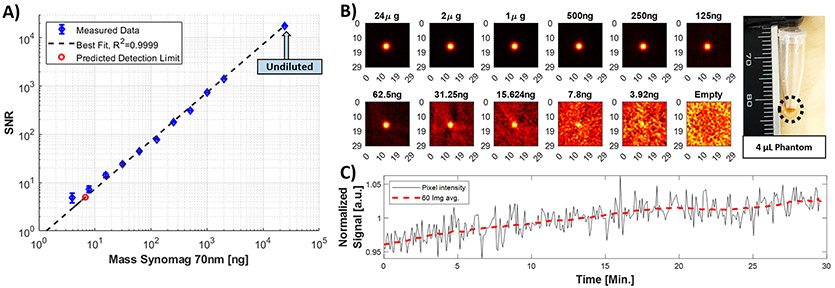
Sensitivity measurements from a dilution series of Synomag-D 70nm (Lot:16321104-02) derived from 5 second images. **A)** Image SNR in the 5 sec image as a function of Fe mass in the sample. **B)** Inverse radon reconstructions of each phantom. Photograph of a representative 4.0 *μ*L phantom in its 0.25 mL microcentrifuge tube is seen on the right. **C)** The time-series of 5 second images for a 4.0 *μ*L, 250 ng sample (roughly corresponding to expected Fe concentration in the brain, *in vivo*).

The drift in the drive coil current measured over 30 minutes of continuous imaging (~8 mT peak drive field), starting from cold, is plotted in Figure 9. In that time, the total magnitude drift is about 1%, and after the first 10 minute transient, the rate of drift in current magnitude is −0.011 % per minute, which corresponds to roughly 1 *μ*T peak drift per minute. The phase drift does not show the same initial transient, and is consistently about 0.00059 Radians per minute. The second measure of stability is seen in Figure 10C where a single 250 ng phantom is imaged for 30 minutes (after 10 minutes of imaging to stabilize). In that time the peak image intensity drifts 6.5% pk-pk from the mean intensity over those 30 minutes.

**Figure 11:**
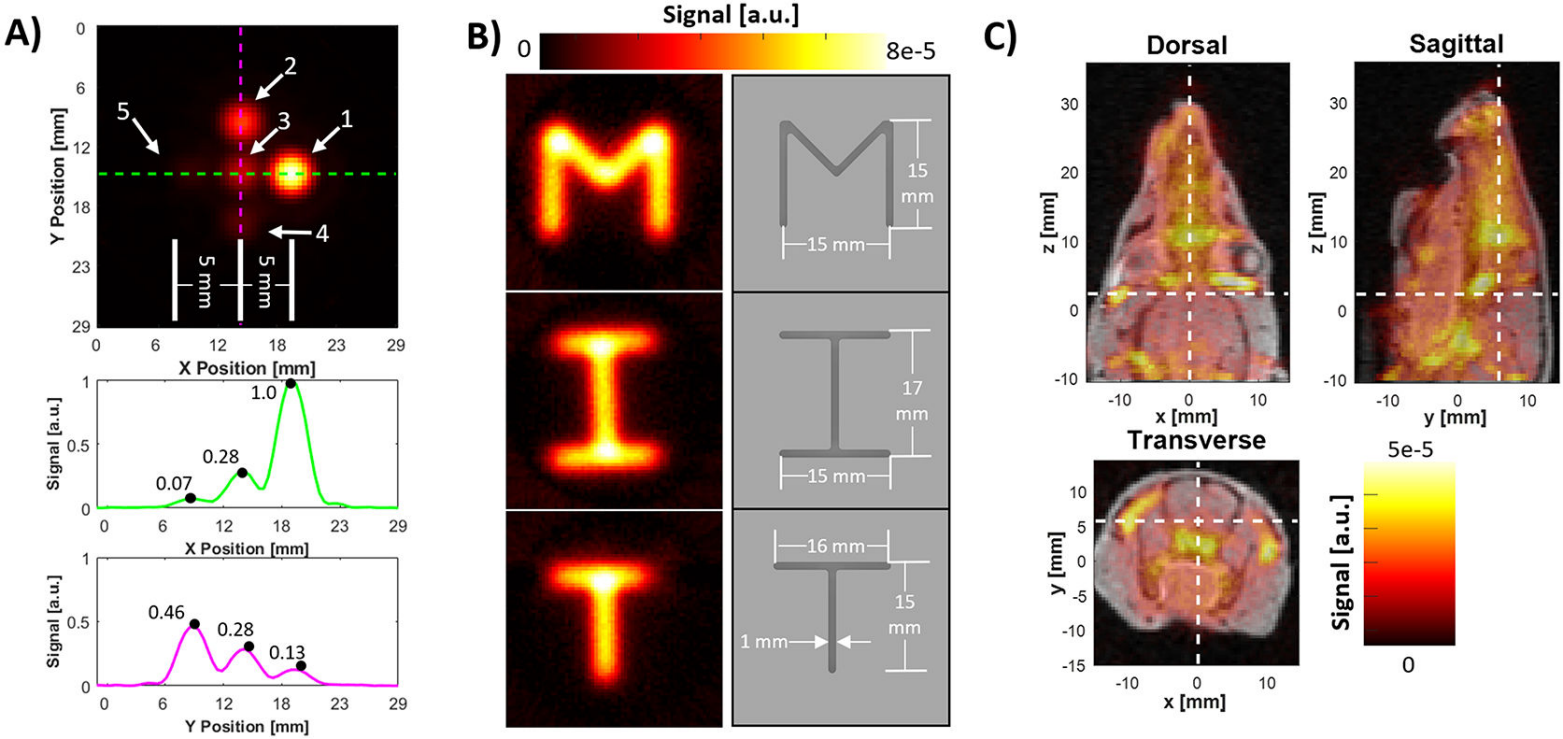
Phantom and *in vivo* results for the scanner, using Synomag-D 70 nm (Lot:16321104-02) and 5 second images. **A)** Five dots on a 5 mm grid (locations marked by arrows) with Fe masses (#1-5): 1000 ng, 500 ng, 250 ng, 125 ng, 62.5 ng respectively. The signal profile, normalized to maximum intensity, is plotted along the green and magenta lines below. **B)** An image of the letters M,I,T filled with Synomag-D 70 nm at a concentration of 0.032 mg Fe/mL. All letters are 1 mm wide, and 1.5 mm deep. **C)** A 3D reconstruction of a rat head coregistered to MRI after injection with (10 mg/kg) SPION. Each transverse MPI slice is a 5 sec image and stacked to form a full 3D volume.

The images in Figure 11 show linearity in a 5-dot phantom with concentrations ranging 16x. We show accurate reconstruction of letter phantoms with concentrations similar to physiological levels (note similar signal amplitudes in Figure 11B and C). We also show *in vivo* imaging data with a volumetric scan of a rat head.

## IV. Discussion

Here, we present an imaging platform capable of stable 5 second imaging continuously for 30 minutes or more (we have used the system for up to 2.5 hours continuously). The system has a detection limit of about 6.7 ng Fe (Synomag-D 70 nm) in a 5 second image, at the target 3 mm FWHM resolution.

### Gantry, shift coils and FFL magnets:

To achieve continuous mechanical rotation of the FFL magnets and shift coils, the system requires slip rings for electrical power and a rotary union for the shift coil water coolant. The feedback controlled gantry is able to rotate at 6 RPM for multiple hours continuously with very little (~1 deg) accumulated rotational error. The choice of mechanically rotating the gantry imposes practical limitations, but the 5 seconds/image is sufficient for functional time-series and comparable to typical fMRI neuroimaging studies.

### Drive and receive chain:

To reach (and surpass) our sensitivity goals, the drive filter topology was designed to have large attenuation (>90 dB) in G_Filter_ above the drive frequency (f_0_) with specific notches at the 2nd and 3rd harmonics (2/3f_0_). It also provides an impedance transformation and common-mode isolation between the drive amplifier and the coil. These features mitigate signal interference due to unstable harmonics from the amplifier and common-mode noise.

While the filter strongly attenuates the harmonics, the noise floor at the harmonic frequencies is still dominated by the instability of insufficiently attenuated amplifier-generated harmonics or harmonics generated by nonlinear effects after the drive amplifier [22]. The narrow-band noise sources are seen as the elevation of 50 kHz and 75 kHz noise levels compared to the surrounding frequencies in Figure 8 when the drive amplifier is on. This is exacerbated by the gradiometer’s rejection ratio which is optimized for 25 kHz but falls off at higher frequencies.

The gradiometer feedthrough attenuation (in Figure 5) shows a clear resonance with maximum suppression at the drive frequency. The frequency of the maximum suppression is determined by parasitic capacitance, the inductive coupling coefficient (K) between drive and receive coils, and other non-idealities causing phase-shift disparities [23]. Thus, by adjusting the positioning, ‘K’ changes and the maximum attenuation can be tuned to the drive frequency. At frequencies higher than the resonance, the capacitive feedthrough dominates and the gradiometer contributes less to suppression of noise or unwanted harmonics from the amplifier. Reducing the stray capacitance (between drive and receive coil) by either increasing the gap between the two or adding a slotted ground-plane (slots to prevent eddy currents from the drive field) could help suppress the nuisance harmonics. Alternatively, reducing the inductance of the coils would also reduce the effect of the stray capacitance (at the expense of drive and receive efficiency).

To achieve the thermal stability for long acquisitions, the drive coil is water cooled and the drive frequency is at a local minimum of magnitude and relatively flat phase region in $G_{\text{filter}}$. The drive current was stable in magnitude and phase within ~1% over 30+ minutes. Because of topology’s efficiency, ambient cooling of the filter components is adequate (inductors ΔT ≈ 15°C). The temperature within the imaging bore is stable at about 10 °C above water temperature, which is necessary for imaging live animals.

### System performance measures:

The system achieved a detection limit of about 6.7 ng in a 5 second image, with a 3 mm spatial resolution using Synomag-D 70 nm (Lot:16321104-02). In the dilution series (Figure 10A/B), the SNR is linear with SPION concentration as expected (R^2^ = 0.999). The FFL hardware configuration produces a spatial point spread function which is 2.3x broader in-plane (X-Y), than in the through-plane (X-Z or Y-Z) image PSF [4]. A more sophisticated reconstruction [24] may be useful to control the through-plane point spread function and deconvolve the in-plane PSF potentially at the cost of SNR.

The temporal resolution in the imaging presented in this manuscript is 5 seconds. This is sufficient for all anticipated experiments as gross CBV changes are generally much slower than that [1, 3]. Stable operation for over 30 minutes of imaging has been demonstrated and has been observed for far longer (~2.5 hours). Figure 9 shows there is a slow drift in current magnitude and phase resulting in the signal drift in Figure 10C, but the amplitude of these drifts are both much slower, and lower amplitude than the expected physiological signals from brain activation. If necessary, a feedback loop could be implemented to facilitate stability.

### IV.I. Future directions

Future hardware improvements are focused on improving the receive coil system, especially reducing the parasitic capacitances between the drive and receive coils. Further, adding an orthogonal receive coil (saddle-shaped) may provide additional information that can be used to improve the reconstruction.

## V. Conclusion

Here, we designed and validated a rodent head-sized FFL-based MPI system that achieved the spatial-temporal resolution and sensitivity goals to enable rodent fMPI neuroimaging experiments and serve as a proof-of-concept platform for fMPI at the human scale. The electrical and mechanical designs, parts lists, and LabVIEW code for this system available under an open-source license (GNU General Public License v3.0 and CERN Open Hardware License v1.2) at OS-MPI.github.io [25].

## Supplementary Material

Drive+TF+Supplemental

## Figures and Tables

**Table 1:** Simulated and measured parameters of the shift coil. The measurement was done at 3 Hz, and the simulation, done in FEMM 4.2, was also at 3 Hz. The FFL was simulated with the default N48 material in FEMM 4.2.

Parameter	Simulated	Measured
Inductance (L_11_, mH)	19.67	19.58
Inductance (L_22_, mH)	19.67	19.27
Inductance (L_12_, mH)	2.94	3.25
Resistance (R_1_, mΩ)	863.1	811.6
Resistance (R_2_, mΩ)	863.1	812.4
Coil sensitivity [mT per Amp]	0.8	1.0
FFL Gradient Z	2.9	2.6
FFL Gradient X	3.1	2.8

**Table 2:** Simulated and measured parameters of the drive coil. The inductance and resistance were measured with an in-house built auto-balancing bridge impedance analyzer, which was calibrated off of an Agilent 4263B LCR meter (Agilent, Santa Clara, CA).

Parameter	Simulated	Measured
Inductance	78 *μ*H	82 *μ*H
Resistance (25 kHz)	290 mΩ	302 mΩ
Coil sensitivity [mT per Amp]	0.337	0.34
