# Supplementary figures and images for: A sensitive, stable, continuously rotating FFL MPI system for functional imaging of the rat brain
Source: Int J Magn Part Imaging. Author manuscript; Available in PMC 2024 Dec 26. (PMC11671131; doi:10.18416/IJMPI.2022.2212001)

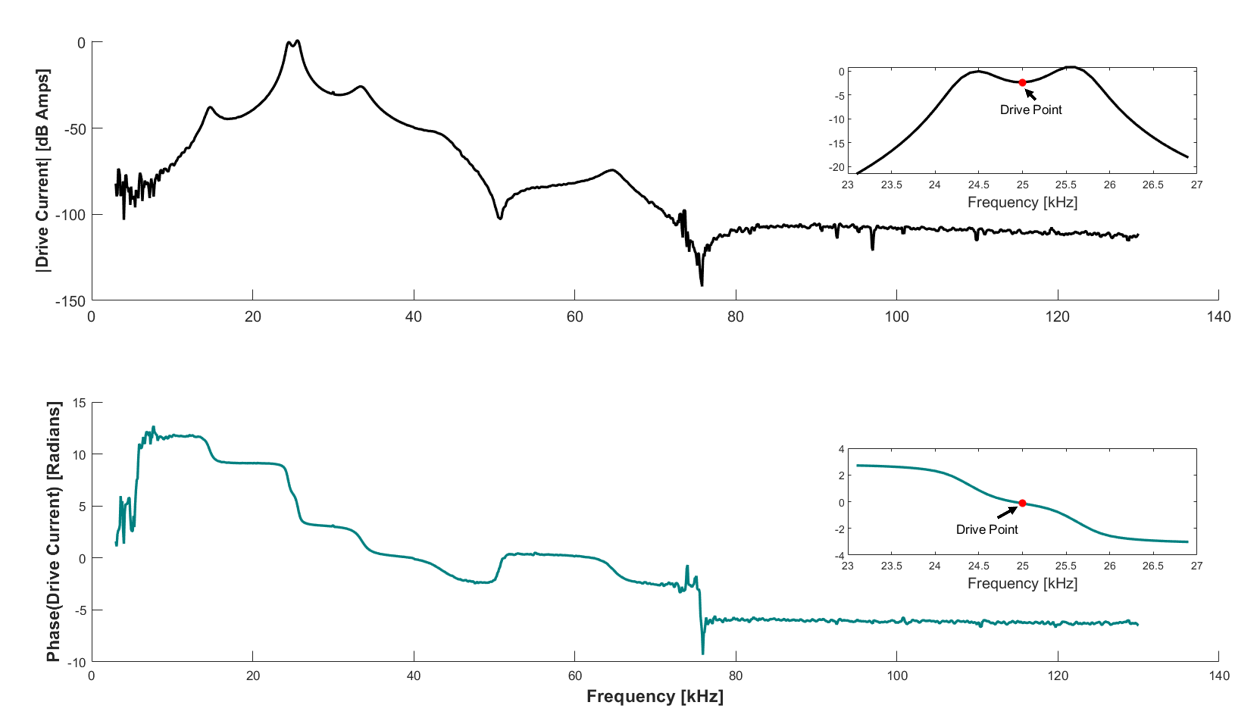

Supplement: Drive+TF+Supplemental [file NIHMS2004181-supplement-Drive_TF_Supplemental.png]
